# The Novel Inflammatory Marker PPN and Systemic Indices as Predictors of Osteoporosis in Postmenopausal Women

**DOI:** 10.1002/kjm2.70168

**Published:** 2026-01-08

**Authors:** Meng‐Yun Pei, Guang‐Ju Long, Peng Zhang

**Affiliations:** ^1^ Graduate School, Shanghai University of Traditional Chinese Medicine Shanghai China; ^2^ Graduate School, Shanghai University of Medicine & Health Sciences Shanghai China; ^3^ School of Public Health, Hainan Medical University Haikou China

Dear Editor,

The pathogenesis of osteoporosis in diabetes mellitus is complex. Current understanding suggests that chronic inflammation, driven by metabolic disorders, plays a key role in the development of both osteoporosis and fragility fractures [1]. Chronic inflammation is also an established factor in postmenopausal osteoporosis [2]. This connection is particularly relevant in diabetes, where chronic inflammation is a known contributor to disease complications. Nevertheless, data on inflammatory markers derived from the complete blood count (CBC) remain limited for postmenopausal women with diabetes aged ≥ 45 years. In this study, we examined a panel of markers that includes not only previously explored indices, such as the monocyte‐to‐lymphocyte ratio (MLR), neutrophil‐to‐lymphocyte ratio (NLR), platelet‐to‐lymphocyte ratio (PLR), aggregate index of systemic inflammation (AISI), and systemic inflammation response index (SIRI), but also two key indices of focus: the polymorphonuclear leukocyte‐to‐platelet ratio (PPN) and the systemic immune‐inflammation index (SII) [3]. We aimed to address this evidence gap by investigating the associations between these inflammatory markers and bone mineral density (BMD), as well as between the markers and the risk of low BMD or osteoporosis [4]. Our analysis focused on subgroups of postmenopausal women with diabetes who were either aged ≥ 65 years or had obesity (BMI ≥ 30 kg/m^2^). We utilized data from four cycles of the National Health and Nutrition Examination Survey (NHANES): 2007–2008, 2009–2010, 2013–2014, and 2017–2018.

The analysis included a total of 501 postmenopausal women with diabetes, all aged 45 years or older. The weighted mean age was 63.70 ± 0.53 years. BMD was measured at multiple hip sites, specifically, the total femur (TF‐BMD), femoral neck (FN‐BMD), trochanter (TR‐BMD), intertrochanter (IN‐BMD), and Ward's triangle (WD‐BMD), using dual‐energy X‐ray absorptiometry (DXA). Inflammatory markers (PLR, MLR, NLR, PPN, AISI, SIRI, and SII) were derived from complete blood count data. Due to their right‐skewed distribution, these markers were log_2_‐transformed for regression analyses and additionally categorized into quartiles to examine trends. To account for the complex survey design and sample weighting, multivariate weighted linear regression and logistic regression models were used to assess associations with BMD and osteoporosis risk, respectively.

Key findings are presented in Table 1. In fully adjusted models, higher levels of log_2_‐transformed inflammatory indices, particularly PPN and SII, were significantly associated with lower BMD at the femoral neck and Ward's triangle in the overall study population. These indices also independently predicted an increased risk of osteoporosis, with odds ratios (ORs) exceeding 2.2 for each 1‐unit increase. These associations were more pronounced in subgroups of women aged ≥ 65 years or with obesity (BMI ≥ 30 kg/m^2^), where the observed effect sizes for both BMD reduction and osteoporosis risk were substantially larger.

**TABLE 1:** Associations between inflammatory indices and hip bone mineral density and osteoporosis risk.

Index	Outcome	Total population			≥ 65‐year‐old subgroup			Obese subgroup (BMI ≥ 30)	
		β/OR (95% CI)	*p*		β/OR (95% CI)	*p*		β/OR (95% CI)	*p*
PPN	FN‐BMD	−0.029 (−0.045, −0.013)	0.001		−0.030 (−0.050, −0.011)	0.003		−0.043 (−0.071, −0.014)	0.003
	WD‐BMD	−0.037 (−0.068, −0.007)	0.018		−0.028 (−0.048, −0.008)	0.006		−0.050 (−0.091, −0.010)	0.016
SII	FN‐BMD	−0.030 (−0.048, −0.012)	0.001		−0.036 (−0.058, −0.015)	0.001		−0.049 (−0.080, −0.019)	0.001
	WD‐BMD	−0.038 (−0.063, −0.013)	0.003		−0.041 (−0.064, −0.018)	0.001		−0.056 (−0.099, −0.014)	0.010
PPN	Ref.log_2_ (PPN)								
	Osteoporosis risk	2.32 (1.31, 4.11)	0.004		2.85 (1.48, 5.51)	0.002		3.12 (1.58, 6.17)	0.001
SII	Ref.log_2_ (SII)								
	Osteoporosis risk	2.35 (1.31, 4.20)	0.004		2.91 (1.55, 5.45)	0.001		3.20 (1.59, 6.42)	0.001
SIRI	Ref.log_2_ (SIRI)								
	Osteoporosis risk	2.33 (1.31, 4.15)	0.004		2.88 (1.55, 5.36)	0.001		3.15 (1.59, 6.25)	0.001
AISI	Ref.log_2_ (AISI)								
	Osteoporosis risk	2.26 (1.35, 3.78)	0.002		2.74 (1.31, 5.71)	0.007		3.13 (1.53, 6.39)	0.002

*Note*: “Ref.log_2_ (index)” denotes a per 1‐unit increase in the log_2_‐transformed inflammatory index. Data are derived from fully adjusted weighted regression models.

Abbreviations: BMD, bone mineral density; FN, femoral neck; WD, Ward's triangle.

Our analysis identifies the PPN as a novel inflammatory predictor, and the SII, SIRI, and AISI as particularly robust predictors of osteoporosis risk in postmenopausal women with diabetes. These findings reinforce the relevance of these systemic indices (SII, SIRI, and AISI) in a high‐risk diabetic population, consistent with earlier studies conducted in broader menopausal contexts [5]. The stronger associations observed in older and obese subgroups underscore the potential clinical utility of these easily accessible, CBC‐derived markers for risk stratification.

## Funding

This work was supported by Xinjiang Uygur Autonomous Region Natural Science Foundation (No. 2022D01A20).

## Ethics Statement

This study involved human participants. The study protocol was reviewed and approved by the Ethics Review Board of the National Center for Health Statistics. All participants provided written informed consent.

## Conflicts of Interest

The authors declare no conflicts of interest.

## Data Availability

The data that support the findings of this study are openly available in NHANES 2007‐2018 at https://wwwn.cdc.gov/nchs/nhanes/Default.aspx.
